# A Fast and Efficient Adaptive Threshold Rate Control Scheme for Remote Sensing Images

**DOI:** 10.1100/2012/691413

**Published:** 2012-11-11

**Authors:** Xiao Chen, Xiaoqing Xu

**Affiliations:** ^1^Jiangsu Key Laboratory of Meteorological Observation and Information Processing, Nanjing University of Information Science and Technology, Nanjing 210044, China; ^2^School of Electronic and Information Engineering, Nanjing University of Information Science and Technology, Nanjing 210044, China

## Abstract

The JPEG2000 image compression standard is ideal for processing remote sensing images. However, its algorithm is complex and it requires large amounts of memory, making it difficult to adapt to the limited transmission and storage resources necessary for remote sensing images. In the present study, an improved rate control algorithm for remote sensing images is proposed. The required coded blocks are sorted downward according to their numbers of bit planes prior to entropy coding. An adaptive threshold computed from the combination of the minimum number of bit planes, along with the minimum rate-distortion slope and the compression ratio, is used to truncate passes of each code block during Tier-1 encoding. This routine avoids the encoding of all code passes and improves the coding efficiency. The simulation results show that the computational cost and working buffer memory size of the proposed algorithm reach only 18.13 and 7.81%, respectively, of the same parameters in the postcompression rate distortion algorithm, while the peak signal-to-noise ratio across the images remains almost the same. The proposed algorithm not only greatly reduces the code complexity and buffer requirements but also maintains the image quality.

## 1. Introduction

A remote sensing image can capture abundant information about geological structures, landforms, and other natural features; therefore, it has been widely applied in meteorology, lunar exploration engineering, environmental monitoring, resource exploration, and other fields. With the rapid development of image sensing technology, the resolution of remote sensing images has gradually increased, and the amount of image data requiring storage and transmission has grown larger. Thus, the real-time data transmission and storage of remote sensing images have become more difficult due to the limited communication bandwidth. For this reason, the fast and effective compression of remote sensing images is crucial. As a new-generation still image compression standard, the JPEG2000 compression algorithm is generally applied to remote sensing images [1–3].

Rate control is an indispensable aspect of the remote sensing image encoding system. It ensures the rational allocation of coded bits to fit the network bandwidth or media storage constraints. Rate control affects not only the bit rate stability but also image quality. Thus, it is important to study effective JPEG2000 rate control algorithms.

Although the JPEG2000 algorithm has excellent compression performance, its standard rate control algorithm is highly complex, thus limiting its application. This complexity derives from embedded block coding with optimized truncation (EBCOT) [4]. EBCOT consists of two phases: Tier-1 encoding and Tier-2 encoding. Tier-1 encoding is an entropy encoding of all of the quantized wavelet coefficients of an image. It accounts for a larger proportion of the computational demands than other parts of the JPEG2000 encoder. The calculation time in this phase consumes 45 to 60% of the total calculation time [5]. The postcompression rate distortion (PCRD) strategy is used for Tier-2 encoding, which establishes the best image quality for a given compressed image storage space. However, the PCRD algorithm is a global optimization process; it searches for the optimal truncation points during Tier-2 encoding after the entire image has been Tier-1-encoded. In addition, the Tier-1 encoding process requires all of the encoded coding passes to be stored, even if only some of the coding passes are included in the final output [6]. Therefore, there are presently many redundant computational steps in the JPEG2000 standard encoding process.

To resolve the shortcomings of the PCRD algorithm, many authors have proposed new rate control algorithms. One proposed solution is a JPEG2000 adaptive rate control algorithm for embedded systems [7]. This algorithm executes JPEG2000 encoding on a set of training images using the PCRD algorithm to determine the linear relationship between the number of subband code passes and the lengths of the corresponding encoding stream bytes. Generating a curve model from this linear relationship can predict the code passes that are included in the final output stream for a given bit rate. A second proposed solution is a new rate adaptive control algorithm for JPEG2000 [8]. The main characteristic of this new control algorithm is its mathematical model of the wavelet coefficients due to the distributive properties of the coefficients in each frequency band after the image wavelet distribution. This model can find the real contribution code rate of every encoded block prior to encoding. When the algorithm computes the plane entropy and every code block reaches the precomputed value, encoding ceases. Therefore, these two algorithms significantly improve the coding efficiency of JPEG2000. However, the experimental results show that the algorithms may lead to a decreased peak signal-to-noise ratio (PSNR) in the recovered image. In a previous study of image recovery, a rate-distortion (*R*-*D*) estimation for fast JPEG2000 compression at low bit rates was developed [9]. However, this estimation utilizes the contexts of the wavelet coefficients, which are typically calculated during Tier-1 encoding; this context generation is a major contributor to the computational complexity of JPEG2000 compression.

In this paper, an improved rate control algorithm based on the bit planes, the *R*-*D* slope, and the compression ratio is proposed. An adaptive threshold formula for Tier-1 encoding is developed. The code that passes below this threshold during Tier-1 encoding is filtered out, thereby avoiding the encoding of all of the code passes. This routine improves the coding efficiency.

## 2. Proposed Rate Control Method

The JPEG2000 standard algorithm consists of the wavelet transform, quantization, and EBCOT coding routines [10, 11]. EBCOT consists of two phases: Tier-1 encoding and Tier-2 encoding. The rate control in the JPEG2000 and PCRD algorithms is performed following the quantization process during Tier-1 and Tier-2 encoding, respectively. Tier-1 encoding is applied once to roughly control the bit rate [12]. Accurate rate control is achieved by the PCRD rate control algorithm during Tier-2 encoding, which selects the coding passes of each code block that are included in the final code stream.

With Tier-1 encoding, most of the computational steps are redundant and waste memory resources. To make better use of the available resources, the characteristics of remote sensing image compression should be examined. On the one hand, as the image compression ratio becomes larger, the target bit rate of the image becomes smaller. When the accumulated bit rate is greater than the target bit rate, the bit plane number is relatively higher, as the bit plane number decreases monotonically. On the other hand, as the image compression ratio becomes larger, the optimal *R*-*D* slope gains also become larger. If we can combine the bit plane number with the compression ratio using a simple formula to calculate the threshold of the *R*-*D* slope adaptively, and if, during the Tier-1 encoding process, we truncate those encoding passes that will be discarded during Tier-2 encoding, the computation and working memory resources required during Tier-1 encoding will be greatly reduced.

In this section, an improved rate control algorithm for remote sensing images is proposed. According to the downward trend in the number of bit planes in the code block and the size of the compression ratio, the threshold of Tier-1 encoding is adaptively adjusted. The proposed rate control method consists of the following steps.

### 2.1. Determination of the Order of the Code Blocks

The larger the number of bit planes required for the code block, the larger the *R*-*D* slope. However, the number of bit planes required for the code block trends roughly downward in the practical encoding process. Therefore, we sort the number of bit planes required for the code block from large values to small values.

Using the value of the number of bit planes required for each code block, we sort the code blocks from the largest bit plane number to the smallest bit plane number, one at a time, after the quantization step during Tier-1 encoding. If two code blocks have the same bit plane number, we sort them according to the original code sequence. In each code block, we encode the passes from the most significant bit plane (MSB) to the least significant bit plane (LSB) by using three coding passes: the significance pass, the refinement pass, and the clean-up pass.

### 2.2. Encoding the Blocks in Which the Accumulation Rate Is Less Than the Target Rate

We calculate the *R*-*D* slope and the accumulation rate of the code passes in each code block, one at a time, after sorting.

The accumulation rate *R* is the sum of the code passes. The *R*-*D* slope of the *j*th code pass is computed according to

(1) $$\begin{array}{c} S_{j}=\frac{D_{j-1}-D_{j}}{R_{j}-R_{j-1}}, \end{array}$$

where *j* is the sequence number of the code pass; *R*_*j*_ and *R*_*j*−1_ are the accumulation rates of *j*th and *j* − 1th passes, respectively; *D*_*j*_ and *D*_*j*−1_ are the distortion of the *j*th and *j* − 1th passes, respectively.

If the accumulation rate is larger than the target rate, then the algorithm proceeds to the next step. Otherwise, this step continues encoding passes of the code blocks.

### 2.3. Finding the Threshold of the *R*-*D* Slope

First, we calculate the minimum number of bit planes required for the code block from each encoded block. Because the code blocks have been sorted downward, the bit plane number of the last encoded block is the minimum bit plane number. Second, we find the minimum slope value *S*_min⁡_ of the encoded blocks by comparing the *R*-*D* slope of the last coding passes of each encoded block. Finally, the threshold of the *R*-*D* slope *λ*_nopt_ is determined by

(2) $$\begin{array}{c} \lambda_{\text{nopt}}\{\begin{array}{cc} S_{\min}, & R_{\max}\leq R<2R_{\max}\\ 2^{\text{numbps}}, & 2R_{\max}\leq R<4R_{\max}\\ 3^{\text{numbps}}, & R\geq 4R_{\max}, \end{array} \end{array}$$

where numbps is the number of bit planes required for the code block and *R*_max⁡_ is the set target rate.

### 2.4. Selection of the Remaining Code Passes

We continue to encode the code passes for which the accumulation rate is greater than or equal to the target rate. Using (1), we calculate the *R*-*D* slope and the accumulation rate of each code pass. If the *R*-*D* slope of this pass is greater than zero and not greater than *λ*_nopt_, it should be discarded, and the remaining code passes will be skipped in this code block. Then, the same processing is performed on the next code block.

When all of the code blocks have been processed, we continue by performing the PCRD algorithm during Tier-2 encoding. The main principle of the PCRD algorithm is to seek the optimal truncation points of each code block within certain bit rate restrictions to minimize distortion.

Using the above steps, we can truncate a large number of code passes that are skipped during Tier-2 encoding by using the threshold *λ*_nopt_. This procedure can greatly reduce the computation and working memory requirements of the image compression. At the same time, the scope of searching for the optimal *R*-*D* slope and the optimal truncation point will be narrowed for Tier-2 encoding.

## 3. Simulation Results and Discussion

In this section, we focus on remote sensing images in the real world and show how our proposed method works.

The proposed rate control algorithm is tested using eight test images that are selected randomly from 30 remote sensing images of different sizes. It is implemented on the Jasper software platform [13], which is defined in Part 5 of the JPEG2000 standard. In each of the images, we use (5, 3) wavelet filters with six-level DWT decomposition with a code block size of 64 × 64 (the default coding parameters in the Jasper software). The simulation results in terms of PSNR are shown in Table 1. The results obtained from using the standard PCRD method are also shown in Table 1. In Table 1, ΔPSNR is defined as

(3) $$\begin{array}{c} \Delta \text{PSNR}=\text{PSN}{\text{R}}_{\text{PROPOSED}}-\text{PSN}{\text{R}}_{\text{PCRD}}. \end{array}$$

Table 1 shows the performance, in terms of PSNR, of the proposed algorithm and the PCRD algorithm. At different bit rates, the PSNR decreases slightly. In most cases, the difference in the PSNR performance is less than 0.1 dB, with the largest difference being 0.151 dB. The proposed algorithm is slightly better than the PCRD algorithm at some bit rates, with improvement in the range of 0.002–0.017 dB. Furthermore, we can see that the PSNR remains unchanged for a bit rate of 1.0 bpp.

**Figure 1:**
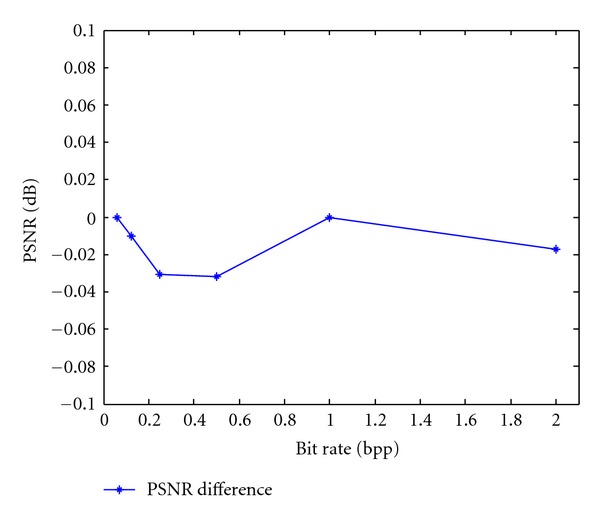
Average PSNR difference between the PCRD and proposed algorithms.

For comparison, Figure 1 shows the average PSNR difference for eight remote sensing images at the different bit rates. From this comparison, we can see that the differences in the average PSNRs are less than 0.04 dB. From Table 1 and Figure 1, we can see that there is little loss of image quality. Therefore, the proposed algorithm can achieve good image quality.

The computation of the algorithm uses the number of Tier-1 code passes to measure the average percentage of computation, defined as

(4) $$\begin{array}{c} \text{Percentage}\;\;\text{of}\;\;\text{computation}=\frac{\text{passe}{\text{s}}_{\text{PROPOSED}}}{\text{passe}{\text{s}}_{\text{PCRD}}}\times 100\%. \end{array}$$

**Figure 2:**
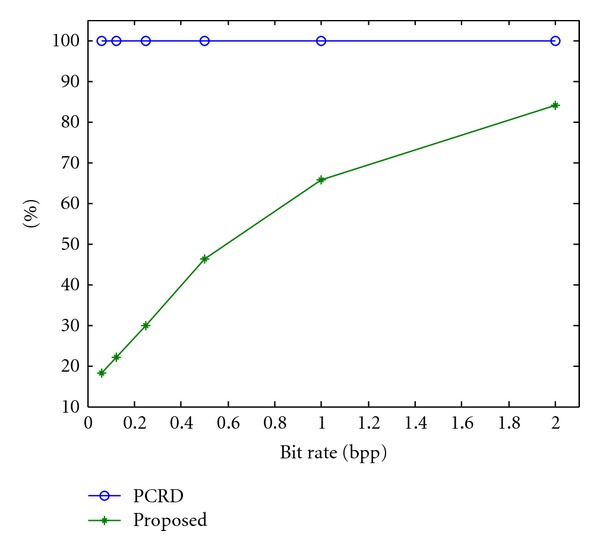
Average computation percentages for the PCRD and proposed algorithms.

Figure 2 shows the average percentage of computation results for remote sensing images undergoing Tier-1 encoding. Compared with the conventional PCRD algorithm, the proposed algorithm can greatly reduce the number of code passes, while the PSNR remains almost unchanged. The proposed algorithm can reduce the computation to 18.13, 22.06, 29.93, 46.23, 65.62, and 83.97% of the computation of the PCRD algorithm at 0.0625, 0.125, 0.25, 0.5, 1, and 2 bpp, respectively.

The memory usage is measured as the number of bytes stored in the memory during Tier-1 encoding. The average percentage of the memory usage is defined as

(5) $$\begin{array}{c} \text{Percentage}\;\;\text{of}\;\;\text{memory}\;\;\text{usage}=\frac{\text{Memor}{\text{y}}_{\text{PROPOSED}}}{\text{Memor}{\text{y}}_{\text{PCRD}}}\times 100\%. \end{array}$$

**Figure 3:**
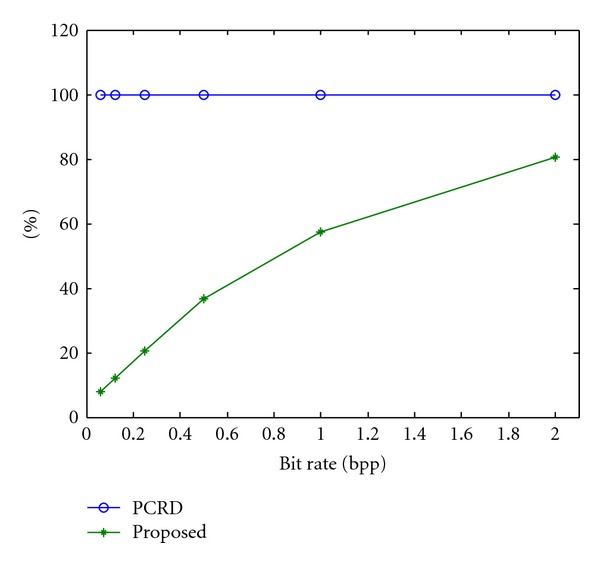
Average memory usage percentages for the PCRD and proposed algorithms.

Figure 3 shows the average percentage of memory usage for the tested images at different bit rates. From the figure, we can see that the memory usage decreases with the decrease in bit rate. The proposed algorithm can reduce the working memory size to 7.81, 12.16, 20.51, 36.54, and 57.53% of the working memory size of the PCRD algorithm at the bit rates of 0.0625, 0.125, 0.25, 0.5, and 1 bpp, respectively. Therefore, the proposed algorithm can greatly reduce the size of the working memory.

**Figure 4:**
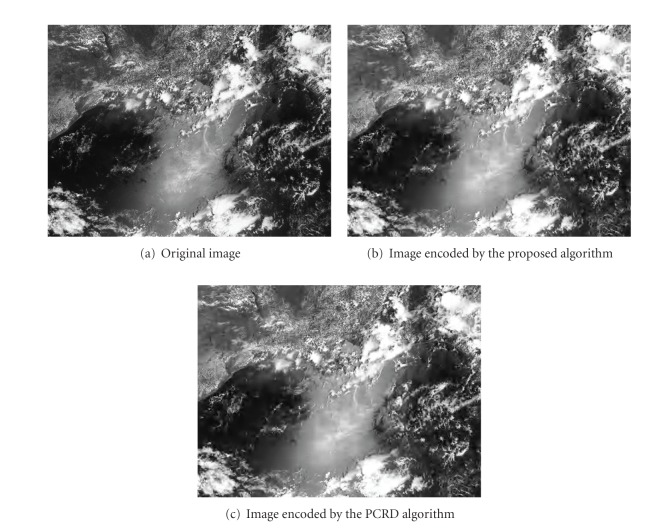
Comparison of the subjective quality of the proposed algorithm and the PCRD algorithm at a bit rate of 0.25 bpp.

To obtain a more intuitive sense of the image quality, Figure 4 shows a comparison of the subjective visual qualities obtained from the proposed and PCRD algorithms at a bit rate of 0.25 bpp and a size of 950 × 712. The two algorithms have the same PSNR and the same subjective quality. However, the computation and the working memory requirements of the proposed algorithm are reduced by nearly 70 and 80%, respectively, according to Figures 2 and 3.

## 4. Conclusion

This paper presents an improved rate control algorithm for remote sensing images. The proposed algorithm puts forward a new adaptive threshold formula for Tier-1 encoding such that the code passes below this threshold during Tier-1 encoding are skipped. Thus, the scope of searching for the optimal *R*-*D* slope threshold and the optimal truncation points is narrowed during Tier-2 encoding. The simulation results show that the proposed algorithm can improve the code efficiency and greatly reduce the buffer size. At the same time, the peak signal-to-noise ratio of coded images remains almost the same.

This paper mainly modifies the rate control of Tier-1 encoding; the next step will be to study the rate control of Tier-2 encoding. If possible, we can combine these two control rates to study a more suitable algorithm for the transmission of a remote sensing image.

## Figures and Tables

**Table 1:** PSNR comparison of the proposed algorithm and the PCRD algorithm.

Test image	PSNR/dB						ΔPSNR/dB				
	Bit rate	0.125	0.25	0.5	1.0	2.0	0.125	0.25	0.5	1.0	2.0
1	PCRD	19.160	20.604	23.080	27.622	36.548	0	−0.151	−0.022	0	0
	proposed	19.160	20.453	23.058	27.622	36.548					
2	PCRD	21.207	22.750	25.086	29.021	35.497	0.008	−0.011	−0.012	0	−0.004
	proposed	21.215	22.739	25.074	29.021	35.493					
3	PCRD	22.532	24.212	26.378	30.096	36.376	0	−0.002	−0.056	0	0
	proposed	22.532	24.210	26.322	30.096	36.376					
4	PCRD	24.742	26.945	29.902	33.904	39.701	0	−0.005	0	0	−0.054
	proposed	24.742	26.940	29.902	33.904	39.647					
5	PCRD	22.303	24.424	27.689	32.748	40.255	0.004	−0.018	0	0	−0.082
	proposed	22.307	24.406	27.689	32.748	40.173					
6	PCRD	17.639	19.449	21.862	25.865	32.024	0	0.017	−0.011	0	0
	proposed	17.639	19.466	21.851	25.865	32.024					
7	PCRD	22.908	24.864	27.303	30.702	36.664	−0.044	−0.022	−0.057	0	0
	proposed	22.864	24.840	27.246	30.702	36.664					
8	PCRD	22.939	24.913	27.399	30.798	36.721	−0.052	−0.052	−0.098	0	0.002
	proposed	22.887	24.861	27.301	30.798	36.723					
